# PD-1 and PD-L1 co-expression predicts favorable prognosis in gastric cancer

**DOI:** 10.18632/oncotarget.19318

**Published:** 2017-07-18

**Authors:** Yanhua Wu, Donghui Cao, Limei Qu, Xueyuan Cao, Zhifang Jia, Tiancheng Zhao, Quan Wang, Jing Jiang

**Affiliations:** ^1^ Division of Clinical Research, First Hospital of Jilin University, Changchun, Jilin 130021, China; ^2^ Department of Pathology, First Hospital of Jilin University, Changchun, Jilin 130021, China; ^3^ Department of Gastric and Colorectal Surgery, First Hospital of Jilin University, Changchun, Jilin 130021, China; ^4^ Department of Endoscopy Center, China-Japan Union Hospital of Jilin University, Changchun, Jilin 130021, China

**Keywords:** gastric cancer, prognosis, PD-L1, tumor infiltrating immune cells, EBV

## Abstract

While the prognosis of gastric cancer (GC) remains poor, PD-1 and PD-L1/L2 are promising prognostic biomarkers. We evaluated PD-1 and PD-L1/L2 expression in tumor cells (TCs) and tumor-infiltrating immune cells (TIICs). We determined the *Helicobacter pylori* (*Hp*) and Epstein-Barr virus (EBV) infection status in a GC cohort (n=340), then analyzed the relationship between the expression of PD-1, PD-L1/L2 and GC prognosis. We found that *PD-1*, *PD-L1*, and *PD-L2* mRNA levels were up-regulated in GC tissues, and were positively correlated with one another (*P=0.043, P=0.008* and *P=0.035*). PD-1 protein expression in TIICs was observed in 22.6% of GC patients. The PD-L1 and PD-L2 positivity rates were 40.3% and 53.8% in TCs, respectively, and 60.0% and 60.9% in TIICs, respectively. PD-L1 was up-regulated in EBV-infected GC patients in both TCs (*P*=0.009) and TIICs (*P*=0.003). *Hp* status was not associated with PD-1 or PD-L1/PD-L2 expression. In TIICs, PD-L1 expression was independently associated with better GC prognosis (HR=0.72, 95%CI: 0.53-0.99). Co-expression of PD-1 and PD-L1, but not PD-L2, was a favorable prognostic marker that indicated a dose effect on the mortality risk of GC patients (*P-*value for trend=0.005). Comprehensive evaluation of PD-1 and PD-L1 in TCs and TIICs could help predict the prognosis of gastric cancers, as well as reveal patients who might benefit from targeted treatment.

## INTRODUCTION

Gastric cancer (GC) is a major cancer-related threat to global health [1, 2]. In 2015, it was the second most common type of cancer, and the third leading cause of cancer deaths in China, accounting for an estimated 339,300 deaths [3]. GC prognosis remains poor because typical symptoms are usually absent in the early stages, resulting in delayed diagnosis and treatment. There is also a lack of reliable biomarkers available for screening targeted therapies and predicting prognosis. Immunotherapy has been considered as an anticancer treatment [4]. The suppression of immune checkpoint pathways may be the most promising approach. This includes the cytotoxic T-lymphocyte–associated protein 4(CTLA-4) and programmed cell death 1(PD-1) pathways [5].

PD-1 and its ligand PD-L1/PD-L2 are a group of negative co-stimulatory molecules that can suppress T cell proliferation in carcinoma [6–8]. The clinical efficacy of PD-1/PD-L1 inhibition has been observed for various malignancies, such as melanoma, non-small cell lung cancer (NSCLC), and renal cell carcinoma (RCC). In addition, clinical trials have shown that positive expression of PD-L1 is associated with a higher response rate to anti-PD-1/PD-L1 treatment [9]. PD-1/PD-L1 might be useful biomarkers for predicting cancer survival. A fraction of patients with PD-L1-negative tumors responded to PD-1 inhibitors in clinical trials [10], suggesting that PD-L2 may predict treatment response.

The stroma of gastric cancer is infiltrated with numerous T lymphocytes [11]. This implies that gastric cancer development is more related to the immune microenvironment than that of other cancers. Therefore, tumor microenvironment-based biomarkers might be more valuable in predicting GC prognosis. PD-L1 and PD-L2 are expressed not only in the tumor cells (TCs), but also in the tumor-infiltrating immune cells (TIICs) [12, 13]. Patients with positive PD-L1 expression in the TIICs have a greater response to anti-PD-1/PD-L1 treatment. The immune stroma-based PD-L1/L2 expression may be clinically valuable. Meanwhile, researchers have begun to follow the relationship between PD-L1 expression in TIICs and cancer prognosis with great interest, but the results have failed to give rise to consistent conclusions [14–16]. Thus, the assessment of PD-L1/L2 expression in TIICs in our cohort may be beneficial in providing new insights into the utility of the PD-1 and PD-L1/L2 pathway in the prediction of GC prognosis.

Infection promotes the susceptibility to, and development of, gastric cancer. *Helicobacter pylori* (*H. pylori; Hp*) and Epstein-Barr virus (EBV) infections are the most common infection-related risk factors of gastric cancer [17, 18]. *H. pylori* infection is positively associated with early inflammatory, precancerous lesions of GC [19]. Similar to *H. pylori*, EBV can stimulate the oncogenic process of GC by promoting chronic inflammation and increased tissue damage [20]. *Hp* and EBV infections promote the formation of the tumor immune microenvironment [21, 22].

A comprehensive molecular classification of gastric adenocarcinomas was reported by The Cancer Genome Atlas (TCGA), and the EBV-positive GC (EBVaGC) accounted for 5.0-17.9% of gastric cancers [23, 24]. EBV-positive GC is characterized by increased levels of genes encoding PD-1, PD-L1, and PD-L2 [24]. *H. pylori-*infected GC and EBV-positive GC may have different states of immunity compared with other types of GC and may lead to an increased PD-1 and PD-L1/L2 expression. In a Western cohort, abundant PD-L1 expression was found in EBV-infected gastric cancers. In a large Asian cohort, PD-1 and PD-L1/L2 expression could not be definitively correlated with *H. pylori*-infected or EBV-positive GC.

In the current study, we evaluated PD-1 and PD-L1/L2 expression in tumor cells (TCs) and tumor-infiltrating immune cells (TIICs). We determined the *Helicobacter pylori* (*Hp)* and Epstein-Barr virus (EBV) infection status in a large GC cohort, then analyzed the relationship between the expression of PD-1, PD-L1/L2 and GC prognosis.

## RESULTS

### Subject characteristics

**Figure 1:**
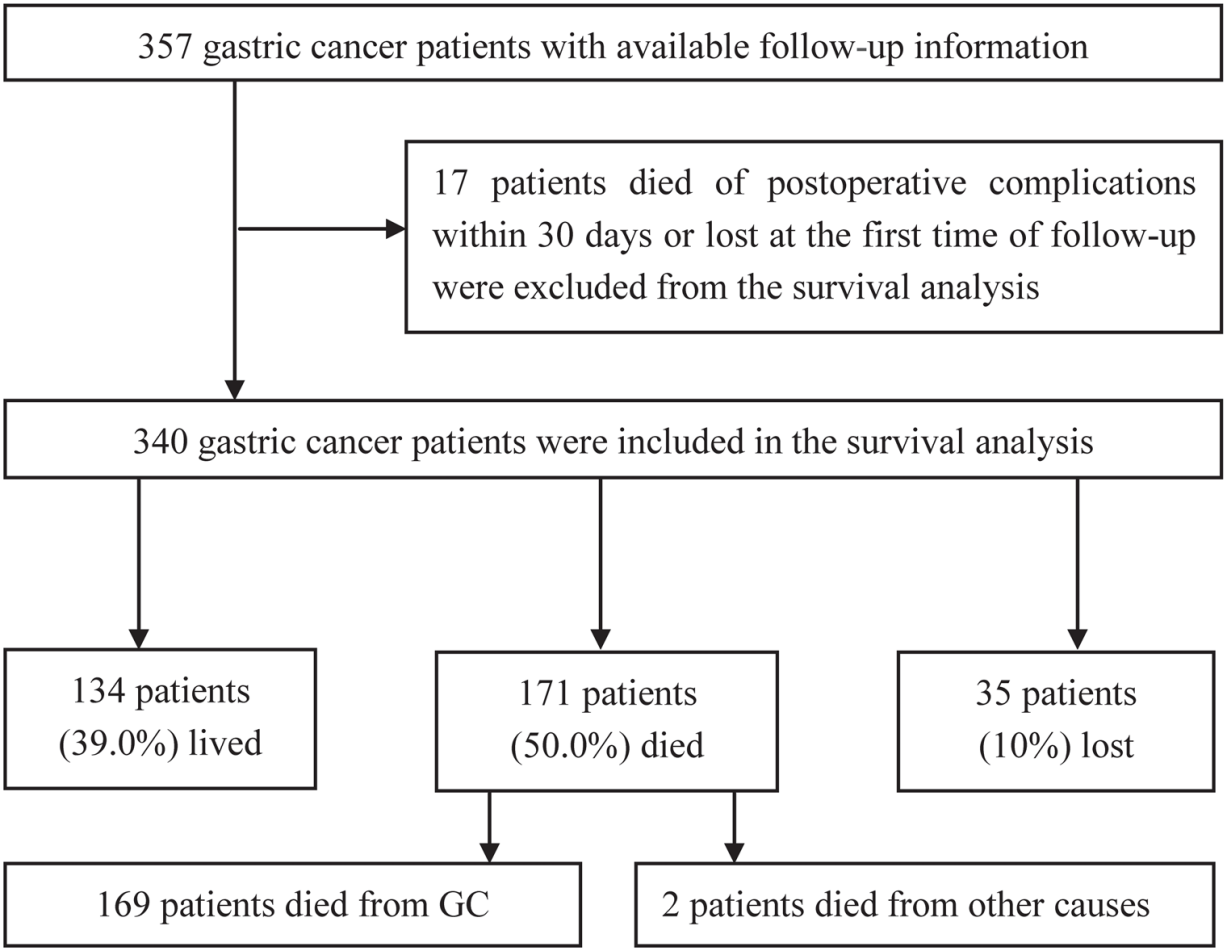
Flow chart of the subjects enrolled

In a total of 357 cases, 6 patients died of postoperative complications within 30 days, and 11 patients were lost to follow-up at the first time point. These 17 patients were excluded from our study, and the remaining 340 gastric cancer patients who underwent surgical resection were included in the final analysis. The median follow-up time was 48 months (ranging from 1 to 111 months). During follow-up, 169 (50.0%) patients died of GC, 2 (0.5%) patients died from other causes, 134 (39.0%) patients were still alive, and 35 (10.0%) patients were lost to follow-up (Figure 1). There were 254 (74.7%) males in our study, and the median age was 62 (range 26–87) years. The other characteristics of the GC patient cohort are summarized in Table 1.

**Table 1:** Characteristics of the GC patients

Characteristics	N (%)
Characteristics	N (%)
Gender	
Male	254(74.7)
Female	86(25.3)
Age	
≤45	22(6.5)
>45	318(93.5)
EBV	
Positive	17(5.0)
Negative	323(95.0)
*Helicobacter pylori*	
Positive	62(60.8)
Negative	40(39.2)
WHO classification	
Tubularadenocarcinoma	244(71.8)
Signet ring cell	36(10.6)
Other	60(17.6)
Histological grade	
low grade	89(26.2)
high grade	251(73.8)
Tumor size	
<5cm	118(34.7)
≥5cm	222(65.3)
Vascular invasion	
Negative	73(21.5)
Positive	267(78.5)
Neural invasion	
Negative	124(36.5)
Positive	216(63.5)
Depth of invasion	
T1/T2	34(10.0)
T3/T4	306(90.0)
Lymph metastasis	
N0	65(19.1)
N1/N2/N3	275(80.9)
Distant metastasis	
M0	325(95.6)
M1	15(4.4)
TNM stage	
I – II	96(28.2)
III-IV	244(71.8)
Chemotherapy	
None	234(68.8)
XELOX^a^	12(3.5)
FLOFOX^b^	8(2.4)
Other^c^	86(25.3)
Gender	
Male	254(74.7)
Female	86(25.3)
Age	
≤45	22(6.5)
>45	318(93.5)
EBV	
Positive	17(5.0)
Negative	323(95.0)
*Helicobacter pylori*	
Positive	62(60.8)
Negative	40(39.2)
WHO classification	
Tubularadenocarcinoma	244(71.8)
Signet ring cell	36(10.6)
Other	60(17.6)
Histological grade	
low grade	89(26.2)
high grade	251(73.8)
Tumor size	
<5cm	118(34.7)
≥5cm	222(65.3)
Vascular invasion	
Negative	73(21.5)
Positive	267(78.5)
Neural invasion	
Negative	124(36.5)
Positive	216(63.5)
Depth of invasion	
T1/T2	34(10.0)
T3/T4	306(90.0)
Lymph metastasis	
N0	65(19.1)
N1/N2/N3	275(80.9)
Distant metastasis	
M0	325(95.6)
M1	15(4.4)
TNM stage	
I – II	96(28.2)
III-IV	244(71.8)
Chemotherapy	
None	234(68.8)
XELOX^a^	12(3.5)
FLOFOX^b^	8(2.4)
Other^c^	86(25.3)

a, a combination of capecitabine and oxaliplatinat least three cycles; b, a combination of 5-fluorouracil, leucovorin, and oxaliplatinat least three cycles; c, other chemotherapy at least three cycles.

### PD-1, PD-L1 and PD-L2 expression in TCs and TIICs, according to *H. pylori* status and EBV status

**Figure 2:**
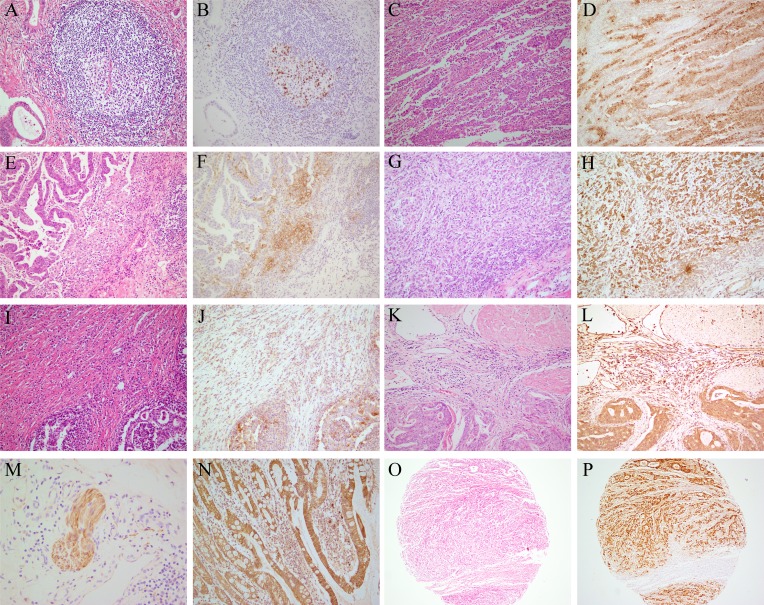
PD-1, PD-L1, and PD-L2 expression in TCs and TIICs of GC patients Routine H&E staining **(A, C, E, G, I, K** and **O)**. **(B)** Positive PD-1 expression in the lymph follicles (IHC). **(D)** Positive PD-L1 expression in tumor cells with a ‘diffuse type’ phenotype (IHC). **(F)** Positive PD-L1 expression at the interface between tumor and non-neoplastic tissues (IHC). **(H)** Positive PD-L2 expression in the tumor cells (IHC). **(J)** Positive PD-L1 expression in the TIICs (IHC). **(L)** Positive PD-L2 expression in the TIICs (IHC). **(M)** Positive PD-L1 expression in the neoplastic nerve fibers (IHC). **(N)** Positive expression of PD-L2 in the intestinal metaplasia of gastric epithelium (IHC). **(P)** Positive detection of EBV in tumor microarrays of gastric cancer (IHC). Magnification: 200× (A-L), 400× (M-N), and 100× (O-P).

PD-1 expression was observed in the TIICs of 77 (22.6%) gastric cancer patients, especially in the lymph follicles (Figure 2A, 2B). PD-L1 and PD-L2 expression was observed in both TCs and TIICs (Figure 2C to 2L). Membranous and cytoplasmic expression was both regarded as positive expression. In TCs, the IRS values for PD-L1 and PD-L2 expression both ranged from 0 to 6 (median 2 for PD-L1; median 1 for PD-L2). A total of 137 (40.3%) patients were positive for PD-L1 in TCs, and 183 (53.8%) patients were positive for PD-L2 in TCs. For the TIICs, the prevalence of positive PD-L1 and PD-L2 expression was 60.0% and 60.9%, respectively. PD-L1 expression patterns in TCs were divided into ‘diffuse type’ (Figure 2C, 2D) and ‘interface type’ (Figure 2E, 2F). We observed PD-L1 overexpression in the neoplastic nerve fibers (Figure 2M), and PD-L2 overexpression in the intestinal metaplasia of the gastric epithelium (Figure 2N).

*H. pylori* infection status data were available for 102 patients, and *Hp* infection was found in 62 (60.8%) cases. There were no associations between the expression of PD-1, PD-L1, or PD-L2 and *Hp* status (Table 2). EBV status was evaluated in all 340 patients, and the prevalence of EBV infection was 5.0% (see Figure 2O, 2P). Positive PD-L1 expression in the TCs was more frequent in EBV-infected GC patients (*P<*0.01). EBV-infected GC patients also had greater PD-L1 expression in the TIICs (*P<*0.01). We found no association between the expression of PD-1 and PD-L2 with EBV status (Supplementary Figure 1, Table 2).

**Table 2:** PD-1 and PD-L1/L2 expression in TCs and TIICs, according to *H. pylori* status and EBV status

	ALL N=340	*H. pylori status*		*P*	EBV status		*P*
		Positive(n=62)	Negative(n=40)		Positive(n=17)	Negative(n=323)	
PD-1 in TIICs							
Positive	77(22.6%)	15(24.2%)	10(25.0%)	0.926	5(29.4%)	72(22.3%)	0.494
Negative	263(77.4%)	47(75.8%)	30(75.0%)		12(70.6%)	251(77.7%)	
PD-L1 in TCs							
Positive	137(40.3%)	24(38.7%)	19(47.5%)	0.380	12(70.6%)	125(38.7%)	**0.009**
Negative	203(59.7%)	38(61.3%)	21(52.5%)		5(29.4%)	198(61.3%)	
PD-L1 in TIICs							
Positive	204(60.0%)	36(58.1%)	26(65.0%)	0.484	16(94.1%)	188(58.2%)	**0.003**
Negative	136(40.0%)	26(41.9%)	14(35.0%)		1(5.9%)	135(41.8%)	
PD-L2 in TCs							
Positive	183(53.8%)	24(38.7%)	21(52.5%)	0.171	7(41.2%)	176(54.5%)	0.283
Negative	157(46.2%)	38(61.3%)	19(47.5%)		10(58.8%)	147(45.5%)	
PD-L2 in TIICs							
Positive	207(60.9%)	29(46.8%)	24(60.0%)	0.192	10(58.8%)	197(61.0%)	0.858
Negative	133(39.1%)	33(53.2%)	16(40.0%)		7(41.2%)	126(39.0%)	

### Clinicopathological characteristics associated with the expression of PD-1, PD-L1, and PD-L2 in TCs and TIICs

PD-1 expression was more prevalent in the younger patients (≤45) than in the older patients (*P*=0.002), and it was more prevalent in patients with smaller tumors (diameter<5 cm) than in those with larger tumors (*P*=0.024) (Table 3). In TCs, the prevalence of positive PD-L1 expression was higher in patients with tubular adenocarcinoma, tumors with diameters larger than 5 cm, invasive depth of T3/T4, absence of lymph metastasis and lower TNM stage (*P*<0.05). PD-L1 expression in TIICs was positively correlated with males, tumor type of tubular adenocarcinoma, larger tumor size, tumor with neural invasion, and lower TNM stages (*P*<0.05) (Table 4). No associations were found between PD-L2 expression and clinicopathological characteristics in either TCs or TIICs (Supplementary Table 1).

**Table 3:** Clinicopathological characteristics of patients according to the expression of PD-1

Characteristics	PD-1 positive	PD-1 negative	*P*
	(N=77)	(N=263)	
Gender			
Male	60(23.6%)	194(76.4%)	0.460
Female	17(19.8%)	69(80.2%)	
Age			
≤45	11(50.0%)	11(50.0%)	**0.002**
>45	66(20.8%)	252(79.2%)	
WHO classification			
Tubular adenocarcinoma	51(20.9%)	193(79.1%)	0.124
Signet ring cell	13(36.1%)	23(63.9%)	
Other	13(21.7%)	47(78.3%)	
Histological grade			
low grade	20(22.5%)	69(77.5%)	0.963
high grade	57(22.7%)	194(77.3%)	
Tumor size			
<5cm	35(29.7%)	83(70.3%)	**0.024**
≥5cm	42(18.9%)	180(81.1%)	
Vascular invasion			
Negative	16(21.9%)	57(78.1%)	0.867
Positive	61(22.8%)	206(77.2%)	
Neural invasion			
Negative	31(25.0%)	93(75.0%)	0.432
Positive	46(21.3%)	170(78.07%)	
Depth of invasion			
T1/T2	11(32.4%)	23(67.6%)	0.154
T3/T4	66(21.6%)	240(78.4%)	
Lymph metastasis			
N0	13(20.0%)	52(80.0%)	0.571
N1/N2/N3	64(23.3%)	211(77.4%)	
Distant metastasis			
M0	75(23.1%)	250(76.9%)	0.378
M1	2(13.3%)	13(86.7%)	
TNM stage			
I-II	24(25.0%)	72(75.0%)	0.516
III-IV	53(21.7%)	191(78.3%)	

**Table 4:** Clinicopathological characteristics of patients according to the expression of PD-L1

Characteristics	PD-L1 positivein TCs (N=137)	PD-L1 negativein TCs (N=203)	*P*	PD-L1 positivein TIICs(N=204)	PD-L1 negativein TIICs(N=136)	*P*
Gender						
Male	109(42.9%)	145(57.1%)	0.091	162(63.8%)	92(36.2%)	**0.014**
Female	28(32.6%)	58(67.4%)		42(48.8%)	44(51.2%)	
Age						
≤45	5(22.7%)	17(77.3%)	0.082	9(40.9%)	13(59.1%)	0.059
>45	132(41.5%)	186(58.5%)		195(61.3%)	123(38.7%)	
WHO classification						
Tubular adenocarcinoma	108(44.3%)	136(55.7%)	**0.002**	154(63.1%)	90(36.9%)	**<0.001**
Signet ring cell	5(13.9%)	31(86.1%)		10(27.8%)	26(72.2%)	
Other	24(40.0%)	36(60.0%)		40(66.7%)	20(33.3%)	
Histological grade						
low grade	34(38.2%)	55(61.8%)	0.640	52(58.4%)	37(41.6%)	0.724
high grade	103(41.0%)	148(59.0%)		152(60.6%)	99(39.4%)	
Tumor size						
<5cm	31(26.3%)	87(73.7%)	**<0.001**	61(51.7%)	57(48.3%)	**0.023**
≥5cm	106(47.7%)	116(52.3%)		143(64.4%)	79(35.6%)	
Vascular invasion						
Negative	29(39.7%)	44(60.3%)	0.911	34(46.6%)	39(53.4%)	**0.008**
Positive	108(40.4%)	159(59.6%)		170(63.7%)	97(36.3%)	
Neural invasion						
Negative	52(41.9%)	72(58.1%)	0.640	74(59.7%)	50(40.3%)	0.927
Positive	85(39.4%)	131(60.6%)		130(60.2%)	86(39.8%)	
Depth of invasion						
T1/T2	8(23.5%)	26(76.5%)	**0.036**	18(52.9%)	16(47.1%)	0.376
T3/T4	129(42.2%)	177(57.8%)		186(60.8%)	120(39.2%)	
Lymph metastasis						
N0	34(52.3%)	31(47.7%)	**0.028**	43(66.2%)	22(33.8%)	0.260
N1/N2/N3	103(37.5%)	172(62.5%)		161(58.5%)	114(41.5%)	
Distant metastasis						
M0	132(40.6%)	193(59.4%)	0.574	196(60.3%)	129(39.7%)	0.590
M1	5(33.3%)	10(66.7%)		8(53.3%)	7(46.7%)	
TNM stage						
I-II	50(52.1%)	46(47.9%)	**0.005**	67(69.8%)	29(30.2%)	**0.021**
III-IV	87(35.7%)	157(64.3%)		137(56.1%)	107(43.9%)	

### Survival analysis of PD-1 and PD-L1/L2 pathway

Tumor size ≥5 cm, positive vascular invasion, and positive neural invasion were associated with poor GC prognosis (Supplementary Table 2). Patients with depth of invasion of T1/T2 had a longer OS than patients with depth of invasion of T3/T4 (log-rank *P*=0.027). Patients without lymph metastasis or any distant metastasis lived longer than patients with lymph metastasis or any distant metastasis (*P*<0.001 and *P*=0.003). Overall, patients with TNM stages I-II had better survival than those with TNM stages III-IV (*P*<0.001).

**Figure 3:**
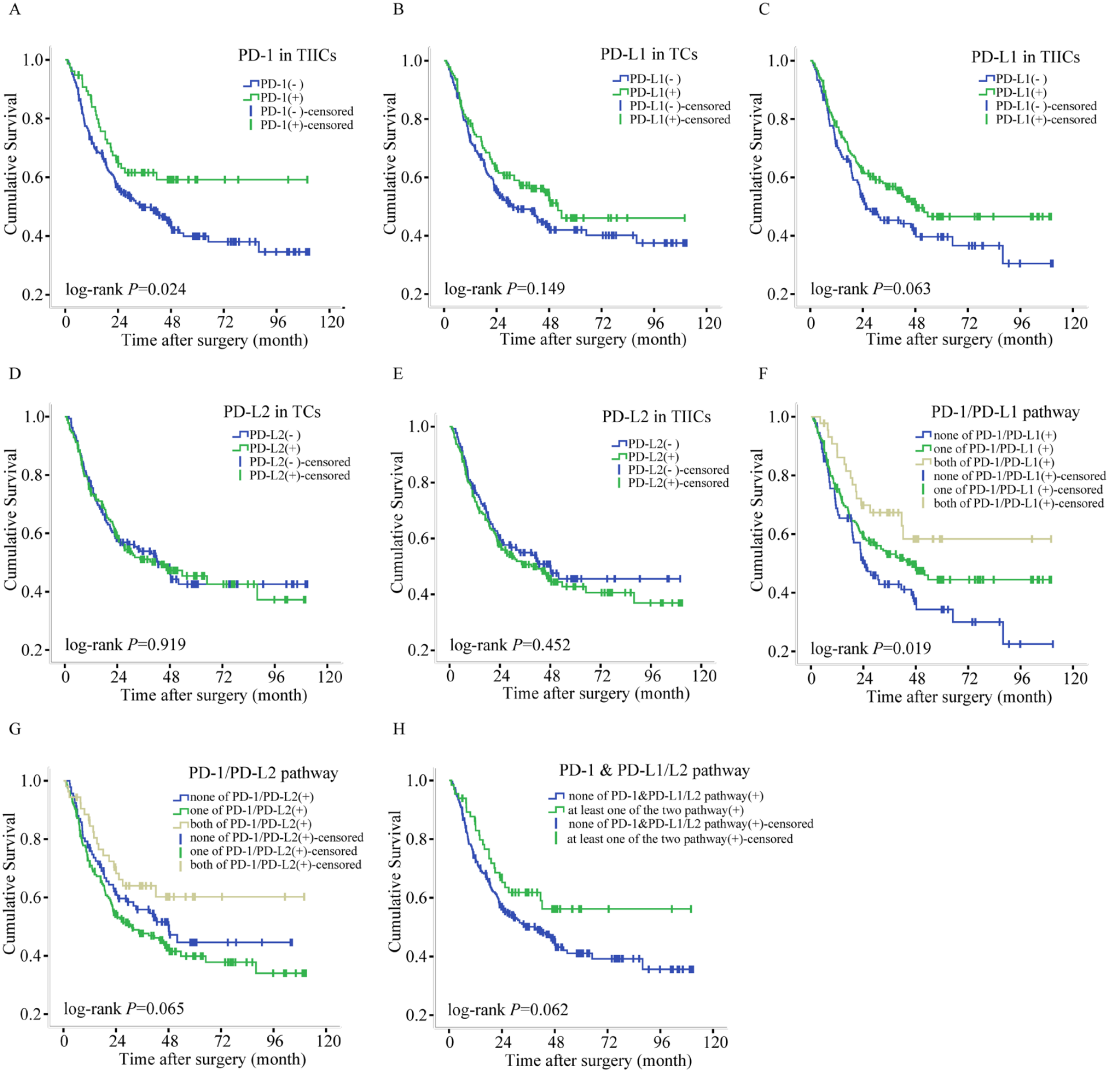
Survival plots for PD-1, PD-L1, and PD-L2 expression of gastric cancer **(A)** Survival plots for PD-1 in TCs of gastric cancer. **(B)** Survival plots for PD-L1 in TCs of gastric cancer. **(C)** Survival plots for PD-L1 in TIICs of gastric cancer. **(D)** Survival plots for PD-L2 in TCs of gastric cancer. **(E)** Survival plots for PD-L2 in TIICs of gastric cancer. **(F)** Survival plots for PD-1/PD-L1 pathway of gastric cancer. **(G)** Survival plots for PD-1/PD-L2 pathway of gastric cancer. **(H)** Survival plots for PD-1 & PD-L1/L2 pathway of gastric cancer.

In univariate analysis, patients expressing PD-1 showed a better overall survival rate (log-rank *P*=0.024). The overexpression of PD-L1 in the TIICs appeared to be associated with better overall survival, although it did not reach statistical significance (log-rank *P*=0.063). PD-L2 expression in TCs and TIICs was not associated with overall survival (log-rank *P*=0.919 and *P*=0.452). Tumors were defined as overall positive for PD-L1 if PD-L1 was expressed in either TCs or TIICs. The same rule was applied to PD-L2. Patients with PD-1/PD-L1 co-expression had better prognosis (log-rank *P*=0.019). Patients with PD-1/PD-L2 co-expression also had better overall survival, although this did not reach statistical significance (log-rank *P*=0.065) (Figure 3 and Table 5).

**Table 5:** Univariate and multivariate Cox regression test for PD-1 and PD-L1/L2 expression

Characteristics	Patient(N)	Death N (%)	HR(95%CI)	*P*	HR(95%CI)^a^	*P*^a^
PD-1 in TIICs						
Negative	263	143(54.37)	**1.00**	**0.024**	1.00	0.052
Positive	77	29(37.66)	**0.63(0.42-0.94)**		0.67(0.45-1.00)	
PD-L1 in TCs						
Negative	203	110(54.19)	1.00	0.150	1.00	0.075
Positive	137	62(45.25)	0.80(0.58-1.09)		0.74 (0.53-1.03)	
PD-L1 in TIICs						
Negative	136	76(55.88)	1.00	0.064	**1.00**	**0.042**
Positive	204	96(47.06)	0.75(0.56-1.02)		**0.72(0.53-0.99)**	
PD-L1 overall						
Negative	124	69(55.64)	1.00	0.094	1.00	0.058
Positive	216	103(47.69)	0.77(0.57-1.05)		0.74(0.54-1.01)	
PD-L2 in TCs						
Negative	183	92(50.27)	1.00	0.919	1.00	0.820
Positive	157	80(50.96)	1.02(0.75-1.37)		0.97(0.71-1.31)	
PD-L2 in TIICs						
Negative	133	64(48.12)	1.00	0.452	1.00	0.342
Positive	207	108(52.17)	1.13(0.83-1.53)		1.16(0.85-1.59)	
PD-L2 overall						
Negative	117	56(47.86)	1.00	0.352	1.00	0.366
Positive	223	116(52.02)	1.16(0.85-1.60)		1.16(0.84-1.60)	
PD-1/PD-L1 pathway						
none of PD-1/PD-L1(+)	91	55(60.43)	1.00		1.00	
one of PD-1/PD-L1 (+)	204	101(49.51)	0.74 (0.53-1.03)	0.071	**0.70(0.50-0.99)**	**0.043**
both of PD-1/PD-L1(+)	45	16(35.56)	**0.47(0.27-0.83)**	**0.009**	**0.48(0.27-0.84)**	**0.010**
PD-1/PD-L2 pathway						
none of PD-1/PD-L2(+)	92	45(48.91)	1.00		1.00	
one of PD-1/PD-L2 (+)	195	108(55.38)	1.22(0.86-1.73)	0.257	1.20(0.84-1.70)	0.323
both of PD-1/PD-L2(+)	53	19(35.85)	0.71(0.41-1.21)	0.203	0.75(0.44-1.28)	0.289
PD-1 & PD-L1/L2 pathway						
none of PD-1& PD-L1/L2 pathway(+)	274	146(53.28)	1.00		1.00	
at least one of the two pathway(+)	66	26(30.39)	0.67(0.44-1.02)	0.064	0.68(0.45-1.04)	0.076

HR: Hazard ratio ^a^: 95%CI and *P* values were calculated with multivariate Cox regression with the enter method including the variables that *P*<0.05 from the univariate analysis, such as tumor size, vascular invasion, neural invasion and TNM stage.

Multivariate Cox regression showed that PD-1 expression in TIICs seemed to be correlated with better survival, but this association did not reach statistical significance (Table 5; Hazard Ratio (HR)=0.67, 95%CI: 0.45-1.00, *P*=0.052). PD-L1 expression in TIICs was independently associated with better GC prognosis (HR=0.72, 95%CI: 0.53-0.99, *P*=0.042). The co-expression of PD-1 and PD-L1 was independently associated with better prognosis of GC, and indicated a dose-effect on the mortality risk of cancer patients (*P*-value for trend=0.005). Compared to the group with no PD-1/PD-L1 expression, the HR of the group with either PD-1 or PD-L1 expression was 0.70 (95%CI=0.50-0.99, *P*=0.043). In the PD-1/PD-L1 co-expression group, the HR was 0.48 (95%CI=0.27-0.84, *P*=0.010). PD-L2 was not independently associated with GC prognosis, in either TCs or TIICs (*P*>0.05). The presence of at least one of the PD-1/PD-L1 or PD-1/PD-L2 combinations was positively connected and almost correlated with better survival, but without statistical significance (HR=0.68, 95%CI: 0.26-1.00, *P*=0.076). Additionally, a higher TNM stage was always independently associated with worse prognosis of GC (*P*<0.001, data not shown).

### *PD-1* and *PD-L1/L2* mRNA expression

**Figure 4:**
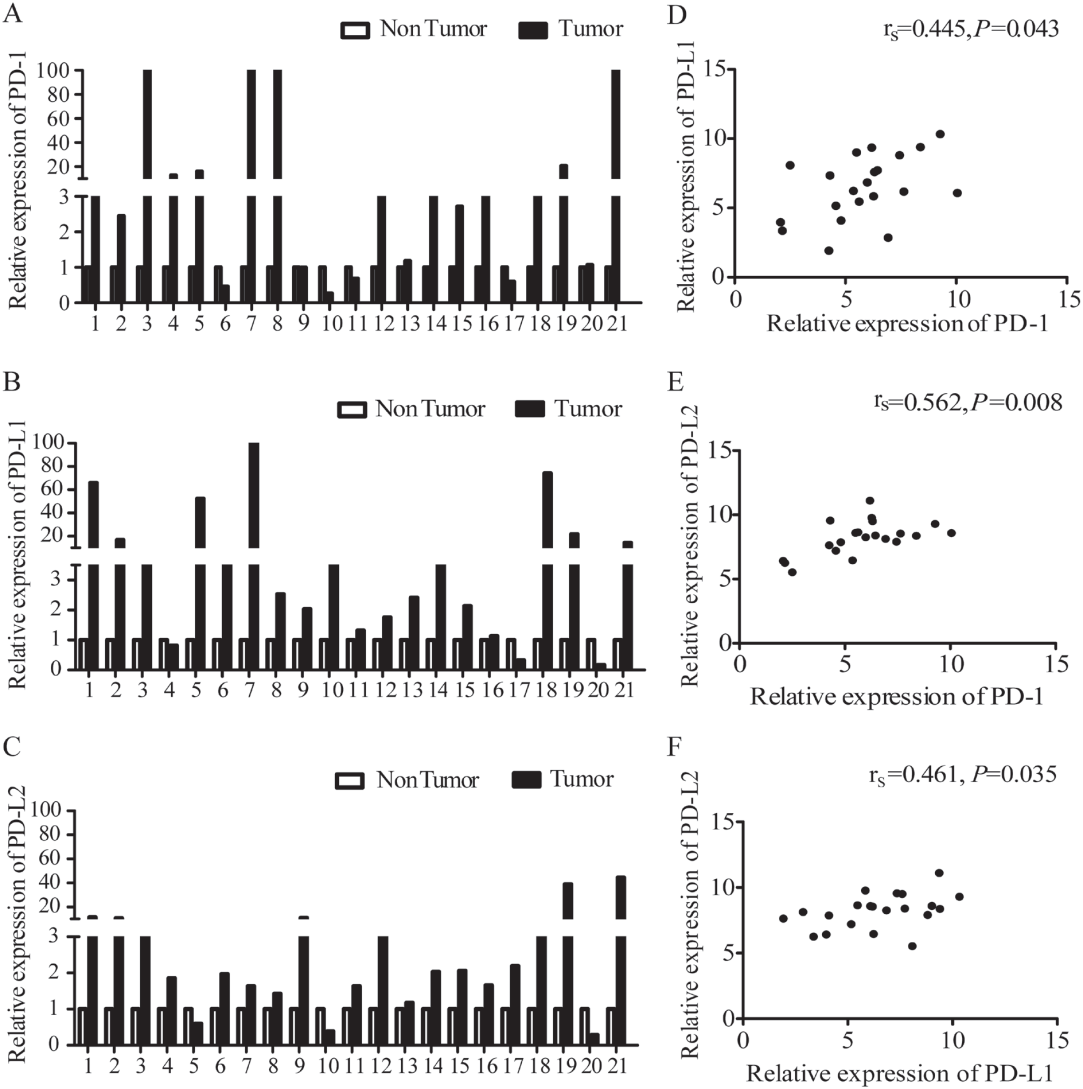
Expression of *PD-1* and *PD-L1/L2* mRNA **(A)** *PD-1* expression in GC tumors and paired adjacent tissues (located > 3 cm away from the tumor). **(B)** *PD-L1* expression in GC tumors and paired adjacent tissues. **(C)** *PD-L2* expression in GC tumors and paired adjacent tissues. **(D)** Correlation of mRNA levels of *PD-1* and *PD-L1*. **(E)** Correlation of mRNA levels between *PD-1* and *PD-L2*. **(F)** Correlation of mRNA levels between *PD-L1* and *PD-L2*. r_s_ represents Spearman's correlation coefficient.

Among the 340 GC patients for whom IHC staining was conducted, twenty-one GC patients were randomly selected from the ‘EBV positive’ (7 cases) and ‘EBV negative’ (14 cases) groups to determine *PD-1, PD-L1*, and *PD-L2* mRNA expression by qRT-PCR. The overall expression level of *PD-1* was increased in 16 (76.19%) GC samples (Figure 4A). *PD-L1* and *PD-L2* were both up-regulated in 18 (85.71%) GC samples (Figure 4B and 4C), which indicates that the expression of *PD-1*, *PD-L1* and *PD-L2* mRNA was frequently up-regulated in gastric cancer tissues. We also found that the expression of *PD-L1* and *PD-L2* was positively correlated with *PD-1* expression (r_s_=0.445, *P*=0.043 and r_s_=0.562, *P*=0.008, respectively). *PD-L2* expression also increased as *PD-L1* expression increased (Figure 4D to 4F). To confirm our results, we analyzed mRNA levels of the same genes in 444 gastric cancer and 32 normal samples from the TCGA database. Consistent with our results, the *PD-1*, *PD-L1*, and *PD-L2* mRNA levels were up-regulated in the GC tissues compared to the normal samples (*P*=0.010, *P*<0.001 and *P*=0.018, respectively) and positively correlated with one other (Supplementary Table 3).

Unlike the protein levels, the *PD-L1* mRNA levels did not display significant differences between the EBV-positive and EBV-negative patients (*P*=0.582). To better understand this, we next analyzed the correlation between *PD-L1* mRNA and protein levels. We found that *PD-L1* mRNA expression in the PD-L1 protein-positive group was not different from that in the PD-L1 protein-negative group (Supplementary Figure 2).

## DISCUSSION

The present study was performed on a large and well-characterized cohort to simultaneously evaluate the expression of PD-1, PD-L1, and PD-L2 in TCs and TIICs of GC patients. PD-L1 was over-expressed in EBV-infected GC patients. The levels of *PD-1*, *PD-L1*, and *PD-L2* mRNA were up-regulated in GC tissues and positively correlated with each other. The co-expression of PD-1 and PD-L1 was found to be a favorable prognostic factor in gastric cancer.

A meta-analysis of 10 studies showed PD-L1 in the TCs of 25% to 65% of Asian (especially in Japanese and Chinese) patients [25], similar to our results (40.3% in TCs). As the TCGA revealed, EBV-positive GC might have PD-L1 gene amplification, and EBV-positive GC has often been characterized by a marked lymphoid infiltration and higher immune response [24, 26]. Considering these findings, EBV-positive GC might have higher PD-L1 expression. In fact, PD-L1 was more frequently expressed in EBV-infected GC cases in both TCs and TIICs in our study, consistent with another study in a Japanese cohort [27]. However, no difference in *PD-L1* mRNA level was found between the EBV-positive and EBV-negative patients. Over 70% of EBV-infected GC patients did not receive any radiotherapy or chemotherapy after surgery. EBV-positive patients may benefit from the PD-L1 targeted therapy due to up-regulated PD-L1 expression, but the EBV infection itself had limited value in predicting the prognosis of gastric cancer.

We found that PD-L1 protein and mRNA levels were not correlated in gastric cancer. This phenomenon has also been observed in breast cancer [28]. One possible reason could be that the testing for *PD-L1* mRNA is always done simultaneously on TCs and TIICs. However, the expression of PD-L1 protein in the TCs and TIICs are evaluated separately and combined as simply positive expression or negative expression. Another possible reason could be that there is some post-transcriptional regulation taking place. Similar to the mRNA expression, biomarkers that expressed in tumor cells or immune cells could not be distinguished by western blot. Western blot also presented a trend that aligned with the IHC results, especially in the patients with positive expression of PD-L1 both in TCs and TIICs (Supplementary Figure 3).

*Helicobacter pylori* strains were enriched among the cagPAI pathogenicity gene variants in Asian GC patients [29]. A recent study reported that the expression of PD-L1 could be up-regulated through *H. pylori* type 4 secretion system (T4SS) and CagA protein in *H. pylori*-infected gastric cancer cell lines [30]. We also compared the levels of PD-L1 mRNA in *H*. *pylori* positive and *H*. *pylori* negative GC. Using the data from the TCGA, we found that there was no association between the level of *PD-L1* mRNA and *H*. *pylori-*infected gastric cancer (Supplementary Table 4).

We determined that PD-L1 expression in the TIICs was an independent predictive biomarker in the prognosis of GC, and was associated with better OS. The same result was found in a German cohort [30] and Japanese cohort [27]. However, the meta-analysis reported that PD-L1 expression was associated with poor OS in GC. PD-L1 is expressed in TCs and TIICs [31–33], but most of the studies included in the meta-analysis only assessed PD-L1 expression in TCs.

PD-L1 expression in TIICs can have prognostic value for other cancers, especially in those tumors that are enriched with infiltrating immune cells [34, 35]. PD-L1 expression in TCs and TIICs might involve different mechanisms. A recent transcriptome analysis reported that PD-L1 expression in TCs could be driven by tumor-intrinsic mechanisms, such as the activation of endogenous oncogenes and related signaling pathways [36]. However, PD-L1 expression in TIICs is up-regulated through adaptive mechanisms, including exogenous inflammation-mediated immune attack, which indicates preexisting immunity [35, 37]. PD-L1 expression in TIICs has a stronger relationship with the cancer immune response, and it depends on the tumor microenvironment compared with the tumor-based PD-L1.

Gastric cancer is an inflammation-related disease, and infiltration of tumor-specific T cells has been observed in the process of GC development. Previous studies have shown that the expression of PD-L1 in TIICs was positively correlated with the number of CD4^+^ T lymphocytes and CD8^+^ T lymphocytes [38], and a high level of expression of CD4^+^ and CD8^+^ T cells has been correlated with better cancer survival [36, 39]. In addition, our results showed that the positive expression of PD-L1 was more common at the interface between the tumor and non-neoplastic tissues and was more frequently seen in patients with TNM stages I-II; thus, to some extent, the expression of PD-L1 indicated an effective immune response in the early stages of cancer progression. Second, most of the mentioned studies have used tumor tissue microarrays (TMAs) to conduct IHC staining. However, TMAs only contain a limited amount of tissue samples (diameter 0.6-2 mm), which result in poor representation of the tumor. The expression of PD-L1 in GC displays intra-tumoral heterogeneity. A recent study investigated the concordance rate of PD-L1 IHC scores between two TMAs and found that the scores were frequently discordant with each other (the discord an cerate was 83.2% in TCs and 65.3% in TIICs) [27]. If only TMAs are used to conduct the IHC staining, there might be a higher risk of non-representation, leading to a higher rate of false-negative results. Furthermore, TMAs are usually constructed with the tumor core with a high concentration of tumor cells. However, tumor inflating immune cells are usually concentrated at the invasion front or stroma, rather than at the central part of tumors. As a result, TMAs are not suitable for evaluating biomarkers that are expressed in TIICs. To solve these problems, we used slides with whole tumor sections to assess the expression of PD-L1 in TCs and TIICs to improve the accuracy of testing. Third, the scoring systems that are used for the assessment of IHC staining are varied. When assessing the expression of PD-L1/L2 in tumor cells, we used the immunoreactive score (IRS) to evaluate the IHC staining. IRS is a semi-quantitative evaluation system and considers the percentages of the stained cells together with the staining intensity. In addition, the percentages of the stained cells were categorical and could be non-uniform, as most of the slides showed low percentages of PD-L1/L2 positive stained cells. Thus, in our opinion, IRS was the appropriate method for testing the expression of PD-L1/L2 in TCs. Additionally, different cutoff values for distinguishing the positive and negative expression of PD-L1 might cause heterogeneity among different studies. Similar to study of Christine et al [30], we used an IRS score of more than 2 as the positive cutoff point when assessing the positive expression of PD-L1 and PD-L2 in TCs. The expression of PD-L1 in TIICs was mainly limited to weak or moderate staining intensity; hence, only the presence or absence of staining was evaluated in most studies that investigated the PD-L1 expression in TIICs [40–42]. Since the minimal expression of PD-L1 might have no effect on tumor biology [30], in our study, “≥5% positive” was classified as “PD-L1-positive in TIICs”.

Though PD-L1 was more frequently expressed in EBV-positive GC patients and PD-L1 positive expression was related to a better survival of GC in our study, EBV infection was not associated with GC prognosis. Further analysis for the clinicopathological features of EBV positive patients indicated that, over 70% EBV infected GC patients not received any radiotherapy or chemotherapy after surgery. In addition to this, only 17 EBV positive GC patients were included in our study, and lead limited statistical power in performing the survival analysis between EBV positive and negative groups.

Through multivariate analysis, we found that PD-1 and PD-L1 co-expression was independently associated with a better prognosis of gastric cancer. Similar results have been observed in a recent study of cutaneous angiosarcoma [43]. The qRT-PCR results also showed that *PD-L1* mRNA levels were positively correlated with *PD-1* mRNA levels. This suggested that PD-1 and PD-L1 may be co-expressed in cancers. PD-L1 has been associated with increasing numbers of CD4^+^ and CD8^+^ T cells [38], both of which express PD-1 [43]. Thus, the binding of PD-1 and PD-L1 might indicate an effective immune response, especially with a favorable immune microenvironment profile [44].

Although the ongoing clinical trials have indicated that PD-L1-positive tumors show higher response rates to anti-PD-1/PD-L1 therapy, good responses to treatment have also been observed in patients with PD-L1-negative tumors [45]. This suggests that besides PD-L1, PD-1 could also bind to other ligands such as PD-L2, and might have effects on the tumor immune response. Our results indicated that PD-L2 was up-regulated in gastric cancer, and this same trend was observed in the TCGA database. We found that overexpression of PD-L2 in TCs or TIICs was not related to the prognosis of GC.

The relationship between PD-L2 expression and cancer prognosis remains controversial. Ohigashi et al [46] reported the positive expression of PD-L2 to be associated with worse prognosis in esophageal cancer patients and in hepatocellular carcinoma [47]. However, the majority of studies have found no correlations between PD-L2 expression and prognosis in other cancers, such as pancreatic cancer, ovarian cancer and pulmonary squamous cell carcinoma, similar to our findings [48–50]. The distinct expression and regulation patterns could partly explain the different predictive values of PD-L1 and PD-L2. First, PD-L1 is widely expressed in a variety of immune cells. When an organism receives strong inflammatory signals, the non-immune cells are also able to up regulate PD-L1 [51, 52]. Compared to PD-L1, PD-L2 expression is much more restricted to the antigen-presenting cells, including macrophages and dendritic cells [53]. This indicates that PD-L1 is more comprehensive in reflecting the immune microenvironment of cancer due to its broader expression. Second, PD-L1 and PD-L2 are differentially regulated by T helper 1 (Th1) and T helper 2 (Th2) cells. In the context of antitumor immunity, Th1 responses are more potent, especially in early stages of tumor progression. Th1/Th2 balance is disrupted as the number of Th2 cells increase, and Th2 responses appear to strengthen tumor immune escape. P'ng Loke et al [54] reported that Th1 cells induce PD-L1 expression, whereas Th2 cells induce PD-L2 expression. Although PD-L2-targeted immune therapy has clinical effects in cancer, the prognostic value of PD-L2 seems to be lower than that of PD-L1.

One limitation of our study is that only a few patients could provide blood samples for the detection of *H. pylori* infection for the investigation of the association between the expression of PD-1 and PD-L1/L2 and *H. pylori*-infected GC. This could have affected the level of statistical efficiency. Another limitation was that despite the median follow-up time being over 47 months, only 50% of the patients had died. Therefore, the follow-up period needs to be extended. Our study was based at a single site, and a multi-center study needs to be conducted in the future. The levels and patterns of PD-1 and PD-L1/L2 may change during surgery and treatment, so monitoring those changes may be helpful in predicting tumor recurrence.

## MATERIALS AND METHODS

### Study population

Patients were recruited from the Department of Gastric and Colorectal Surgery in the First Hospital of Jilin University (Changchun, China) from 2007 to 2014. A total of 357 patients who were newly diagnosed with gastric cancer and had undergone a physical tumorectomy for adenocarcinoma of the stomach qualified for the study. Before surgery, none of the patients had received chemotherapy, radiotherapy, or anti-PD-1/PD-L1 treatment. Two independent pathologists confirmed each patient's diagnosis. The principal clinical characteristics included gender, age, WHO classification of the primary tumor, tumor sizes, TNM stage according to AJCC/UICC, 2010 classifications, and chemotherapy status.

All patients underwent follow-up after the tumorectomy in the third month, sixth month, and every year until death or the last scheduled follow-up. The duration from the date of surgery to the date of death or the last successful interview date was defined as the survival time. Patients who died due to complications of the surgical procedure during the perioperative period or were lost at the first time of interview were excluded in the survival analysis. Before enrollment, all participants signed informed consent forms. All of the analyses in our study were performed after surgery. Our study was approved by the Institutional Review Board of the First Hospital of Jilin University.

### Tests of *H. pylori* and Epstein-Barr virus infection

The *Helicobacter pylori* IgG enzyme-linked immunosorbent assay (ELISA) test (BIOHIT HealthCare, Finland) was used to diagnose *H. pylori* infection in the patients’ sera. A value of 30 EIU or more indicated *H. pylori* infection. The tumors were labelled by DNA *in situ* hybridization (ISH-5021, ZSGB-BIO, China) for EBV infection using tissue microarrays (MiniCore, Alphelys, France). Nuclei stained brown indicated EBV infection.

### Immunohistochemistry

Histological sections (4 μm) of 10% formalin-fixed paraffin-embedded tumor specimens of GC patients were used for immunohistochemical (IHC) staining. We have tested different dilutions of antibodies against PD-1, PD-L1/L2 in patients during the preliminary experiment and the optimal IHC dilutions of each antibody had been well determined. From the instructions and related references about the antibodies above, there was no evidence that the antibodies had a neutralizing effect. Finally, the sections were stained with primary monoclonal antibodies against PD-1 (ab52587, dilution: 1/200, Abcam, Cambridge, UK), PD-L1 (E1L3N, dilution: 1/200, Cell Signaling Technology, Cambridge, UK), and PD-L2 (clone #176611, dilution: 1/200, R&D Systems, Minneapolis, USA). After routine dewaxing, the slides were boiled for 2min (20 min for PD-L1) in a pressure cooker in citrate buffer (ethylenediamine tetraaceticacid for PD-L1 without high pressure) for antigen retrieval. Endogenous peroxidase activity was blocked using 3% H_2_O_2_. The sections were pre-treated with 10% normal goat serum (MXB, Fuzhou, China) before the primary antibodies were applied for 90 min at room temperature. The sections were further incubated with horseradish peroxidase-labeled secondary antibody (MXB, Fuzhou, China) for 15 min at room temperature. The signals were visualized with 3, 3-diaminobenzidine (DAB), and then the slides were counterstained with Mayer's hematoxylin.

### Evaluation of PD-1, PD-L1, and PD-L2 expression

The PD-1 staining was assessed in TIICs by two experienced pathologists blinded to the clinical data. A proportion of stained cells ≥5% in TIICs with a membranous staining was considered PD-1 positive. For PD-L1 and PD-L2, expression was predominantly observed in the cell membrane and cytoplasm. The IRS system was used to assess the staining intensity and percentages of the tumor cells. The intensity and expression prevalence was subdivided into four categories each: 0 (no immunostaining; <5% expression), 1 (weak; 5 to 19% expression), 2 (moderate; 20 to 49% expression), or 3 (strong; ≥50% expression), and the percentages were subdivided into four grades: 0 (<5% expression), 1 (5 to 19% expression), 2 (20 to 49% expression) and 3 (≥50% expression). Adding the intensity and percentage scores resulted in IRS values ranging from 0 to 6. A total score of more than 2 was defined as positive expression of PD-L1 or PD-L2 in TCs. For the evaluation of the expression of PD-L1 and PD-L2 in TIICs, the cases were simply scored as negative (<5% expression) and positive (≥5% expression).

### mRNA quantification

Among the 340 GC patients for whom IHC staining was conducted, total mRNA was extracted from 21 patients’ tumor tissues and paired adjacent non-tumorous tissues (located > 3 cm away from the tumor and confirmed by the pathologists with H&E staining). After reverse transcription, cDNAs were amplified in the presence of primers, and *glyceraldehyde 3-phosphate dehydrogenase* (*GAPDH*) was used as the endogenous reference gene. The expression of *PD-1, PD-L1*, and *PD-L2* was quantified using specific primers (Qiagen), and the mRNA levels of these three genes were analyzed with the 2^−ΔΔCt^ method.

### Western blot analysis

The total protein of gastric tumor tissues and paired adjacent non-tumorous tissues was extracted using a mammalian protein extraction kit (Kangwei, China), and the concentration of various proteins was measured using a BCA kit (Kangwei, China). The levels of PD-1 (1:200, Abcam), PD-L1 (1:2000, Cell Signaling Technology), PD-L2 (1:500, R&D Systems), and GAPDH (1:1000, Abcam) were measured with ECL reagents (Thermo Fisher Scientific) using Molecular Imager Chemi Dox XRS+ imaging system (Biorad, California, USA).

### Statistical analysis

Categorical variables were represented as frequency (percentage) and compared using the χ^2^ test. Continuous variables with normal distribution were represented as the mean ± standard deviation, and compared by Student's t-test. Continuous variables with non-normal distribution were represented by the median (Q1-Q3), and compared using Wilcoxon's rank-sum test. The log-rank test was used to compare Kaplan-Meier survival curves. Univariate and multivariate Cox regressions were performed to assess the hazard ratios (HRs) and 95% CIs of the possible prognostic factors. The correlation between PD-1, PD-L1, and PD-L2 mRNA levels were calculated with Spearman's rank correlation. *P*<0.05 was considered to be statistically significant. All of the analyses were conducted with the SPSS program (version 17.0, Chicago, IL, USA) or GraphPad Prism 5.0.

## CONCLUSION

PD-L1 was over-expressed in EBV-infected GC. The *PD-1*, *PD-L1*, and *PD-L2* mRNA levels were up-regulated in GC tissues and positively correlated with one another. Co-expression of PD-1 and PD-L1, but not PD-L2, was a favorable prognostic marker in gastric cancer. The comprehensive evaluation of tumor cells and tumor-infiltrating immune cells could help in predicting the prognosis of gastric cancers and selecting patients who might benefit from targeted treatment.

## SUPPLEMENTARY MATERIALS FIGURES AND TABLES



## Abbreviations

PD-1: programmed cell death 1
PD-L1: programmed cell death ligand 1
PD-L2: programmed cell death ligand 2
GC: gastric cancer
TCs: tumor cells
TIICs: tumor-infiltrating immune cells
EBV: Epstein-Barr virus
*Hp*: *Helicobacter pylori*
HR: hazard ratio
CI: confidence interval
CTLA-4: cytotoxic T-lymphocyte–associated protein 4
NSCLC: non-small cell lung cancer
RCC: renal cell carcinoma
TCGA: The Cancer Genome Atlas
EBVaGC: EBV positive GC
XELOX: a combination of capecitabine and oxaliplatin
FLOFOX: a combination of 5-fluorouracil, leucovorin and oxaliplatin
IHC: immunohistochemistry
H&E: hematoxylin-eosin
OS: overall survival
T4SS: type 4 secretion system
TMAs: tumor tissue microarrays
IRS: immunoreactive score
Th1: T helper 1
Th2: T helper 2
AJCC/UICC: Union for International Cancer Control/American Joint Committee on Cancer
ELISA: enzyme-linked immunosorbent assay
*GAPDH*: *glyceraldehyde 3-phosphate dehydrogenase*
