# Supplementary material for: PD-1 and PD-L1 co-expression predicts favorable prognosis in gastric cancer
Source: Oncotarget. 2017 Jul 18;8(38):64066–82. doi: 10.18632/oncotarget.19318 (PMC5609984; doi:10.18632/oncotarget.19318)
Supplement: Supplementary file 1 [file oncotarget-08-64066-s001.pdf]

PD-1 and PD-L1 co-expression predicts favorable prognosis in gastric cancer

SUPPLEMENTARY MATERIALS

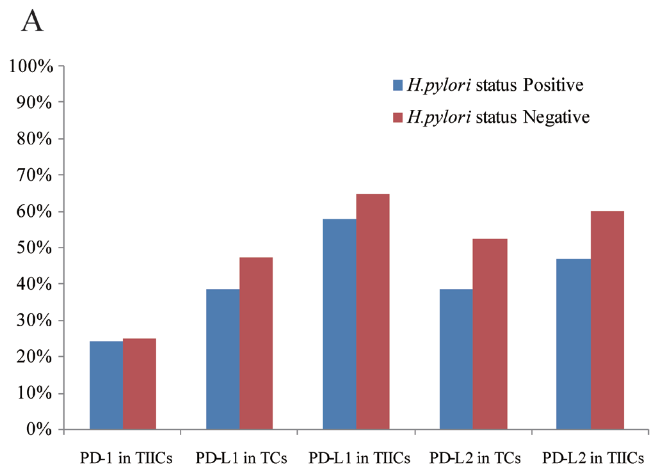

Supplementary Figure 1: Prevalence of PD-1, PD-L1, and PD-L2 expression in TCs and TIICs according to *H. pylori* status and EBV status. **(A)** Prevalence of PD-1, PD-L1, and PD-L2 expression in TCs and TIICs according to *H. pylori* status. **(B)** Prevalence of PD-1, PD-L1, and PD-L2 expression in TCs and TIICs according to EBV status.

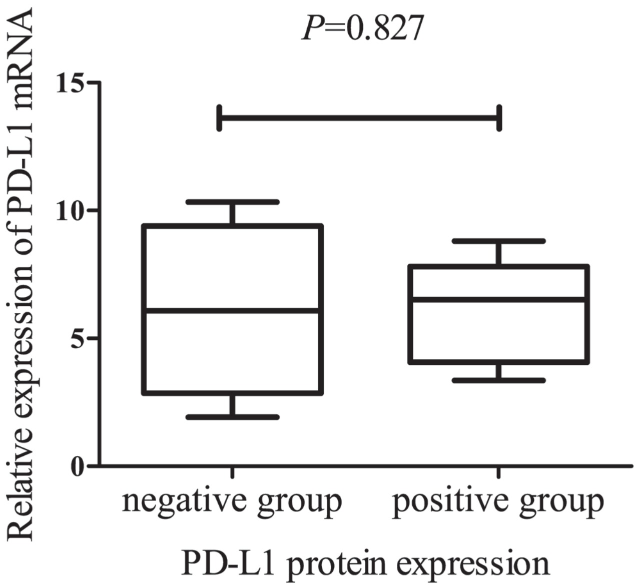

Supplementary Figure 2: Association between the expression of PD-L1 mRNA and protein.

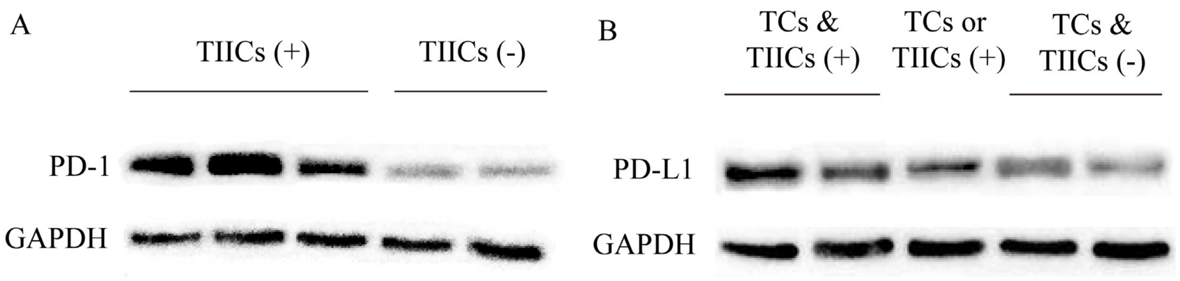

Supplementary Figure 3: Comparison of PD-1, PD-L1/L2 protein levels detected by IHC and western blot.

Supplementary Table 1: Clinicopathological patient characteristics according to PD-L2 expression

Characteristics	PD-L2 positive in TCs (N=183)	PD-L2 negative in TCs (N=157)	*P*	PD-L2 positive in TIICs (N=207)	PD-L2 negative in TIICs (N=133)	*P*
Gender						
Male	133(52.4%)	121(47.6%)	0.353	153(60.2%)	101(39.8%)	0.675
Female	50(58.1%)	36(41.9%)		54(62.8%)	32(37.2%)	
Age						
≤45	13(59.1%)	9(40.9%)	0.608	13(59.1%)	9(40.9%)	0.859
>45	170(53.5%)	148(46.5%)		194(61.0%)	124(39.0%)	
WHO classification						
Tubularadenocarcinoma	128(52.5%)	116(47.5%)	0.571	146(59.8%)	98(40.2%)	0.819
Signet ring cell	19(52.8%)	17(47.2%)		23(63.9%)	13(36.1%)	
Other	36(60.0%)	24(40.0%)		38(63.3%)	22(36.7%)	
Histological grade						
low grade	50(56.2%)	39(43.8%)	0.604	54(60.7%)	35(39.3%)	0.963
high grade	133(53.0%)	118(47.0%)		153(61.0%)	98(39.0%)	
Tumor size						
<5cm	66(55.9%)	52(44.1%)	0.570	73(38.1%)	45(61.9%)	0.787
≥5cm	117(52.7%)	105(47.3%)		134(39.6%)	88(60.4%)	
Vascular invasion						
Negative	37(50.0%)	37(50.0%)	0.456	41(55.4%)	33(44.6%)	0.275
Positive	146(45.1%)	120(54.9%)		166(62.4%)	100(37.6%)	
Neural invasion						
Negative	68(54.0%)	58(46.0%)	0.967	79(62.7%)	47(37.3%)	0.599
Positive	115(53.7%)	99(46.3%)		128(59.8%)	86(40.2%)	
Depth of invasion						
T1/T2	17(50.0%)	17(50.0%)	0.637	21(61.8%)	13(38.2%)	0.912
T3/T4	166(54.2%)	140(45.8%)		186(60.8%)	120(39.2%)	
Lymph metastasis						
N0	34(52.3%)	31(47.7%)	0.785	41(63.1%)	24(36.9%)	0.687
N1/N2/N3	149(54.2%)	126(45.8%)		166(60.4%)	109(39.6%)	
Distant metastasis						
M0	174(53.5%)	151(46.5%)	0.624	198(60.9%)	127(39.1%)	0.943
M1	9(60.0%)	6(40.0%)		9(60.0%)	6(40.0%)	
TNM stage						
I-II	53(55.2%)	43(44.8%)	0.748	64(66.7%)	32(33.3%)	0.170
III-IV	130(53.3%)	114(46.7%)		143(58.6%)	101(41.4%)	

Supplementary Table 2: Survival times of patients with different clinicopathological parameters

Characteristics	Patient (N)	Death N (%)	Median OS (month)	log-rank *P*
Gender				
Male	254	124(48.82)	48.26	0.268
Female	86	48(55.81)	32.00	
Age				
≤45	22	11(50.00)	46.00	0.658
>45	318	161(50.63)	42.22	
EBV				
Positive	17	9(52.94)	41.56	0.824
Negative	323	163(50.46)	43.73	
Helicobacter pylori				
Positive	62	32(51.61)	41.40	0.897
Negative	40	22(55.00)	30.72	
WHO classification				
Tubular adenocarcinoma	244	120(49.18)	46.00	0.161
Signet ring cell	36	21(58.33)	25.40	
Other	60	31(51.67)	48.00	
Histological grade				
low grade	89	38(42.70)	66.89[a]	0.069
high grade	251	134(53.39)	39.00	
Tumor size				
<5cm	118	51(43.22)	67.24[a]	**0.016**
≥5cm	222	121(54.50)	32.00	
Vascular invasion				
Negative	73	25(34.25)	75.65[a]	**0.001**
Positive	267	147(55.06)	30.72	
Neural invasion				
Negative	124	48(38.71)	70.58[a]	**0.001**
Positive	216	124(57.41)	27.11	
Depth of invasion				
T1/T2	34	12(35.29)	75.46[a]	**0.027**
T3/T4	306	160(52.29)	39.56	
Lymph metastasis				
N0	65	14(21.54)	88.62[a]	**<0.001**
N1/N2/N3	275	158(57.45)	27.00	
Distant metastasis				
M0	325	160(49.23)	46.00	**0.003**
M1	15	12(80.00)	13.00	
TNM stage				
I-II	96	27(28.13)	81.23[a]	**<0.001**
III-IV	244	145(59.43)	24.84	
Chemotherapy				
None	234	128(54.70)	32.00	0.065
XELOX	12	5(41.67)	44.83[a]	
FLOFOX	8	5(62.50)	29.44	
other	86	34(39.53)	44.15[a]	

[a] For these characteristics, less than half patients were diead, so mean overall survival (OS) time was presented when median OS could not be calculated.

Supplementary Table 3: Correlation matrix between PD-1, PD-L1, and PD-L2 from TCGA database

	PD-1	***PD-L1***	***PD-L2***
PD-1	1.000	0.615	0.691
PD-L1	0.615	1.000	0.693
PD-L2	0.691	0.690	1.000

Data were shown as spearman r

Supplementary Table 4: Association between PD-1, PD-L1/L2 and *H. pylori* infection from TCGA database

	***H. pylori* positive (n=20)**	***H. pylori* negative (n=157)**	***P***
PD-1	1.44(0.53-2.23)	1.10(0.70-2.14)	0.651
PD-L1	1.18(0.47-3.14)	0.96(0.47-2.00)	0.500
PD-L2	1.16(0.64-2.49)	0.90(0.48-1.75)	0.149

Data were shown as median (Q1-Q3)
